# Sex-specific modulation of T-type voltage-gated calcium channels in the renal artery of hypertensive rats

**DOI:** 10.3389/fphys.2026.1754344

**Published:** 2026-03-16

**Authors:** Andrea Suarez, Sol Guerra-Ojeda, Alicia Valls, David Verdú, Marta Serna-García, Guadalupe Herrera, Eva Serna, Maria D. Mauricio

**Affiliations:** 1 Department of Physiology, InVas and ModulAhR Groups, Facultat de Medicina i Odontologia, INCLIVA Biomedical Research Institute, Universitat de Valencia, Valencia, Spain; 2 Department of Dentistry, Faculty of Health Sciences, Universidad Europea de Valencia, Valencia, Spain; 3 Flow Cytometry Unit, UCIM-IIS INCLIVA, Biomedical Research Institute, Universitat de Valencia, Valencia, Spain; 4 Centro de Investigación Biomédica en Red Fragilidad y Envejecimiento Saludable CIBERFES, Valencia, Spain; 5 Centro de Investigación Biomédica en Red Enfermedades Cardiovasculares CIBERCV, Valencia, Spain

**Keywords:** hypertension, nitric oxide, renal artery, sex differences, T-type VGCCs

## Abstract

**Introduction:**

Hypertension contributes to cardiovascular disease, with growing evidence of sex-specific differences in its underlying mechanisms. T-type voltage-gated calcium channels (VGCCs) have emerged as key regulators of vascular tone, particularly under conditions of nitric oxide (NO) deficiency. However, their role in mediating vascular dysfunction across sexes remains poorly understood.

**Methods:**

This study examined the role of T-type VGCCs and their modulation by NO in the renal artery of male and female spontaneously hypertensive rats (SHR). Vascular reactivity was assessed through phenylephrine-induced contractions in the presence or absence of nickel chloride (NiCl_2_), a T-type VGCC blocker, and L-NAME, an inhibitor of nitric oxide synthase (NOS). Gene expression of T-type VGCCs (Ca_V_3.1 and Ca_V_3.2) and eNOS was quantified by RT-PCR. Oxidative stress parameters in leukocytes were assessed by flow cytometry to explore the systemic redox.

**Results:**

Hypertension induced a rightward shift of the acetylcholine-mediated vasorelaxation curve. In male rats, hypertension did not significantly alter the phenylephrine concentration-response curve. A single data point showed a significant difference following incubation with L-NAME. The contribution of T-type VGCCs to vascular reactivity remained unchanged. Male SHRs displayed increased mRNA expression of Ca_V_3.1, Ca_V_3.2, and eNOS, yet showed no corresponding increase in T-type VGCC activity or NO availability. Conversely, in female SHR, phenylephrine concentration-response curve showed a leftward shift, reduced NO release, and increased participation of T-type VGCCs in response to phenylephrine. Furthermore, pharmacological inhibition of NO synthesis in female WKY rats, used to simulate hypertensive conditions, enhanced the involvement of T-type VGCCs in phenylephrine-induced vasoconstriction. mRNA expression of eNOS was not modified by hypertension in females. Despite the heightened T-type VGCCs activity, female SHRs had reduced mRNA expression of Ca_V_3.1 and Ca_V_3.2, suggesting a potential compensatory downregulation. Finally, leukocytes of male SHR exhibited significantly increased production of H_2_O_2_ and ONOO^−^ compared to the other studied groups, suggesting that hypertension contributes to a greater oxidative stress in male.

**Discussion:**

These findings reveal sex-specific differences in the role of T-type VGCCs during hypertension and underscore the therapeutic potential of targeting T-type VGCCs, particularly in females, as a sex-specific strategy for more effective and personalized hypertension management.

## Graphical Abstract

**Figure:**
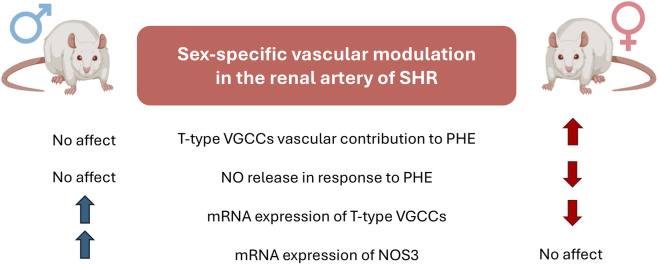
Sex-specific vascular modulation of T-type voltage-gated calcium channels (VGCCs) and nitric oxide (NO) signalling in renal artery of Spontaneously Hypertensive Rats (SHR). Hypertension leads to distinct molecular and functional changes in male and female rat renal arteries. Male rats with hypertension exhibit elevated T-type VGCCs and eNOS mRNA expression (Nos3) without corresponding functional changes in T-type VGCCs activity or NO release in response to phenylephrine (PHE). Female rats with hypertension display enhanced T-type VGCCs functional activity and reduced NO release in response to PHE, with downregulation of T-type VGCCs mRNA. These findings reveal striking sex-specific differences, underscoring the therapeutic potential of targeting T-type VGCCs for personalized hypertension management, particularly in females.

## 1 Introduction

Cardiovascular diseases remain the leading cause of mortality globally, with hypertension representing a growing public health burden. Current estimates from the World Health Organization indicate that hypertension affects approximately 1.3 billion individuals worldwide, contributing to around 9 million deaths annually due to its role in precipitating major cardiovascular events, including myocardial infarction, stroke, and ischemic heart disease (World Health Organization, 2025). Effective blood pressure control significantly reduces such complications, so advancing our understanding of the underlying molecular mechanisms remains imperative for the development of more targeted and tolerable therapies. In this context, experimental models that faithfully reproduce the pathophysiology of hypertension are essential for elucidating these molecular mechanisms and evaluating potential therapeutic strategies. The spontaneously hypertensive rat (SHR) is a well-established model of essential hypertension, as it develops high blood pressure spontaneously without surgical or pharmacological intervention (Doris, 2017; Rizzoni et al., 1994). SHR model exhibits alterations in sympathetic signalling and adrenergic receptor regulation as a consequence of established hypertension (Michel et al., 1990). Adrenergic signalling plays a central role in the regulation of vascular tone (Guimarães and Moura, 2001). Phenylephrine, as a selective α_1-_adrenergic receptor agonist, induces vasoconstriction through the activation of these receptors expressed in vascular smooth muscle. α_1_-adrenergic receptors are not restricted to vascular smooth muscle but are also expressed in endothelial cells of various arteries, where they contribute to vasodilatory responses (McGrath, 2015). Their expression appears low and their functional relevance remains controversial compared with the predominant role of smooth muscle α_1-_adrenoceptors (Guimarães and Moura, 2001). Adrenergic stimulation can lead to increases in endothelial intracellular calcium, thereby promoting the release of vasodilatory factors. However, this effect is generally considered secondary to α_1_-receptor activation in vascular smooth muscle rather than the result of direct α_1_-receptor stimulation in endothelial cells (Guimarães and Moura, 2001; Do et al., 2000). Under physiological conditions, this vasoconstrictor influence is tightly balanced by nitric oxide (NO), that can functionally oppose α_1_-adrenergic contractions, acting as an important counter-regulatory mechanism that preserves vascular homeostasis (Vallance and Chan, 2001).

In hypertension, this delicate balance between adrenergic vasoconstriction and NO-mediated vasodilation is frequently disrupted, resulting in an enhanced α_1_-adrenergic-mediated vasoconstrictor response (Grassi et al., 2015). Concurrently, reduced NO bioavailability—resulting from endothelial dysfunction, oxidative stress, and/or impaired eNOS activity—further amplifies vasoconstrictor responses (Förstermann and Münzel, 2006). The combination of heightened adrenergic signalling and diminished NO-mediated modulation is therefore considered a critical mechanism underlying vascular dysfunction in hypertension.

Beyond the well-established association between hypertension, vascular dysfunction and oxidative stress (Dai and Khalil, 2025; Griendling et al., 2021), dysregulation of calcium signalling also plays an important role. Calcium is an exceptionally versatile signalling molecule, central to a wide range of cellular functions, particularly vascular calcium homeostasis is meticulously regulated by various pathways, including membrane-bound calcium channels that govern ion flux, and intracellular organelles like the sarcoplasmic reticulum and mitochondria that mediate calcium release and reuptake (Bonventre, 1992; Burnham et al., 2002; Clapham, 2007; Nelson et al., 1995; Rapoport et al., 1983). The coordinated action of these cellular mechanisms is indispensable for the autoregulation of blood flow.

Voltage-gated calcium channels (VGCCs) are crucial in maintaining vascular tone. While L-type VGCCs serve as the principal route for calcium influx in vascular smooth muscle cells (VSMCs) (Moosmang et al., 2003), recent research has increasingly highlighted the role of T-type VGCCs (Ca_V_3.1, Ca_V_3.2, and Ca_V_3.3 subtypes) in various vascular beds. Unlike L-type channels, T-type VGCCs activate at lower depolarization thresholds and exhibit faster kinetics, playing a key role in cellular excitability, especially in tissues with rhythmic activity (Todorovic and Lingle, 1998; Weiss and Zamponi, 2019). T-type VGCCs, although contributing modestly to baseline myogenic tone, gain functional prominence in states of oxidative stress or reduced NO signalling (Howitt et al., 2013a; Ng et al., 2024; Smi et al., 2020).

The therapeutic landscape for hypertension is dominated by angiotensin-converting enzyme inhibitors (ACE inhibitors), angiotensin II receptor blockers (ARBs), and calcium channel blockers. In this latter category, L-type calcium channel blockers predominate over T-type blockers, although clinical studies have demonstrated that combined therapy involving both L-type and T-type VGCCs blockers may offer superior efficacy compared to conventional L-type monotherapy, underscoring the growing importance of T-type VGCCs as strategic therapeutic targets (Feng et al., 2004; Koh et al., 2007; Lee et al., 2002; Ohta et al., 2009; Sasaki et al., 2009). Moreover, the blockade of both L-type and T-type VGCCs has been shown to improve endothelial function, an effect not observed when only L-type channels are blocked, according to a recent study in healthy 60-year-old men (Iepsen et al., 2024). Whether this effect also occurs in women remains unknown.

Women tend to exhibit greater antihypertensive responses to diuretics and calcium channel blockers, but are more susceptible to adverse effects from ACE inhibitors. Conversely, men may benefit more from pharmacological agents targeting the renin–angiotensin–aldosterone system, including ACE inhibitors and ARBs (Fan et al., 2008; Rabi et al., 2008; van Luik et al., 2023). These sex-specific differences in drug response and underlying pathophysiological mechanisms underscore the critical need for incorporating sex as a biological variable in hypertension research and treatment strategies. A deeper understanding of how hypertension manifests and progresses differently in men and women is essential for developing more effective, personalized, and equitable therapeutic approaches.

Building upon these observations and acknowledging a critical knowledge gap regarding the sex-dependent regulation of T-type VGCCs in hypertension, this study comprehensively explores their function and expression in the renal artery of both male and female normotensive Wistar Kyoto (WKY) and SHR. We hypothesize that under physiological conditions, NO exerts a downregulatory influence on T-type VGCCs activity; consequently, under conditions of endothelial dysfunction and NO deficiency, such as hypertension, these channels become hyperactive. Given their vasoconstrictor role, this increased activity may exacerbate hypertensive pathology, with distinct manifestations across sexes, mirroring patterns observed for L-type VGCCs (Harada et al., 2025).

In the present study, we focused on the renal artery, a critical site of vascular pathology in hypertension (Sos and Trost, 1995), as it provides the main blood supply to the kidneys. The renal system plays a central role in the long-term regulation of blood pressure through the control of sodium and water balance, extracellular fluid volume, and the pressure-natriuresis relationship. Alterations in renal function can lead to sustained changes in arterial pressure, making the kidney a key determinant in the development and maintenance of hypertension. In addition, the kidney is a major target of neurohumoral mechanisms involved in blood pressure regulation, including sympathetic nervous system activity and the renin-angiotensin-aldosterone system. Dysregulation of these pathways contributes to increased sodium retention, vascular resistance, and impaired pressure natriuresis, all of which are hallmarks of hypertensive pathology (Guyton et al., 1972; Ivy and Bailey, 2014; Wenner et al., 2018). Given this central integrative role, examination of renal involvement is essential for understanding the mechanisms underlying blood pressure dysregulation in hypertension.

Therefore, the overarching aim of this research is to elucidate the role of T-type VGCCs in phenylephrine-induced responses of the renal artery in both normotensive and hypertensive rats, with particular emphasis on modulation by nitric oxide and sex.

## 2 Materials and methods

### 2.1 Experimental animal model

16-week-old male and female SHR and their respective WKY rat controls were obtained from Charles River Laboratories S.A. (Barcelona, Spain) (*n* = 8 per group) and housed in groups of four per cage, separated by sex and experimental group. Animals were maintained at a stable temperature of 23 °C with 12-h light-dark cycles and fed *ad libitum*. We implemented a parallel design to minimize any potential hormonal bias, since oestrous cycle can influence both ion channel activity and eNOS expression, as previously described (Honnens et al., 2011; Saleeon et al., 2015). All animals were purchased from the same supplier at the same age, acclimatized under identical housing conditions, and studied in parallel on the same experimental days. It is well established that female rats housed together tend to synchronize their oestrous cycles through pheromonal signalling (McClintock, 1978), which suggests that the hormonal status of both groups was comparable during the protocols. All experimental procedures were approved by the University of Valencia Ethics Committee (Committee approval number: 2021/VSC/PEA/0264 type 2).

Systolic (SBP), diastolic (DBP), and mean blood/arterial pressure (MAP) were measured non-invasively using the tail-cuff method with a CODA 8 high-throughput system (Kent Scientific Corporation, Torrington, CT, United States) based on Volume Pressure Recording (VPR) technology. To ensure data reliability, rats were acclimated to the restrainers for 15 min daily over three consecutive days before recording. During sessions, animals were placed in holders on a heating platform (32 °C) to ensure adequate tail vasodilation, and parameters were recorded as the average of five daily readings over 2 weeks. Pulse pressure (PP) was calculated as the difference between SBP and DBP. Our measurements confirmed the hypertensive phenotype, as both male and female SHR exhibited significantly higher SBP, DBP, and MAP values compared to their respective WKY controls (*p* < 0.05), while no significant differences were observed in PP between the groups (Supplementary Figure S1). Rats were humanely euthanized by an overdose of the halogenated anaesthetic isoflurane (Isoflo®, ISOVET, B. Braun) delivered at a 5% concentration via inhalation in a rodent induction chamber, following 0.1 mg/kg of buprenorphine analgesia. The depth of anaesthesia was monitored by checking for the lack of response to a noxious mechanical stimulus (firm toe pinch to both fore and hind paws) to ensure a surgical plane of anaesthesia throughout the experiment. Subsequently, a total blood collection was performed via cardiac exsanguination using a 25G intraventricular needle. The blood samples (approximately 2–3 mL) were collected into EDTA-coated tubes to prevent coagulation. A portion of the whole blood was immediately processed for flow cytometry analysis. The renal arteries were dissected for vascular reactivity studies, and some samples were snap-frozen in liquid nitrogen and stored at −80 °C for subsequent gene expression experiments.

### 2.2 Vascular reactivity studies

Renal artery rings 2 mm in length were mounted on a wire myograph (620 M DMT-Danis Myo Technologies) for isometric tension recording utilizing a PowerLab data acquisition system (Model PL8/30, ADInstruments) and LabChart 7 software. Regarding the morphological characteristics of the vessels, the internal diameters of the renal arteries were comparable within each sex. No significant differences were observed between male WKY (442.08 ± 47.98 µm) and SHR (446.44 ± 21.60 µm), nor between female WKY (399 ± 11.76 µm) and SHR (411.67 ± 9.11 µm).

Each ring was immersed in a 5 mL bath containing Krebs-Henseleit solution (composed of 115 mM NaCl, 4.6 mM KCl, 1.2 mM MgCl_2_·6 H_2_O, 2.5 mM CaCl_2_, 25 mM NaHCO_3_, 11.1 mM glucose, and 0.01 mM disodium EDTA). This solution was maintained at 37 °C and equilibrated with a gaseous mixture of 95% O_2_ and 5% CO_2_ to ensure a pH of 7.3–7.4. The basal tension for each myograph-mounted ring was obtained through a normalization based on Laplace’s Law, which was performed using MyoNORM software.

At the beginning of each experimental session, the viability and contractile capacity of each vascular ring were assessed by exposure to a high-potassium solution (KCl 60 mM). The maximal contraction values recorded were 645 ± 233 mg for male WKY, 577 ± 340 mg for male SHR, 390 ± 171 mg for female WKY, and 360 ± 172 mg for female SHR. Concentration-response curves were constructed for phenylephrine (10^–9^ - 3 × 10^−5^ M), acetylcholine (10^–9^ - 10^–5^ M), and sodium nitroprusside (10^–9^ - 3 × 10^−6^ M). Vasoconstrictor responses to phenylephrine were expressed as a percentage of the initial contraction induced by KCl (60 mM), which was considered 100%. In the latter two cases, arteries were previously contracted with noradrenaline (10^–7^ - 10^–6^ M).

To investigate the involvement of T-type VGCCs in the response to phenylephrine, and whether NO modulates the activity of these channels, we calculated the area under the curve (AUC) for the phenylephrine concentration-response curves. We performed the analysis under four different experimental conditions: a control group (without inhibitors or blockers), a group with the T-type VGCC blocker NiCl_2_, a group with the NO synthase inhibitor L-NAME, and a group with both L-NAME and NiCl_2_. Subsequently, ΔAUC was determined by subtracting the AUC of the curve with NiCl_2_ from the control AUC (ΔAUC 1). This value represents the involvement of T-type VGCCs in the absence of L-NAME. We then calculated the ΔAUC between the phenylephrine curve with L-NAME and the curve with L-NAME plus NiCl_2_ (ΔAUC 2). This second value reflects the involvement of T-type VGCCs in the presence of L-NAME. Finally, we compared and plotted ΔAUC 1 and ΔAUC 2 as a bar graph to evaluate the participation of T-type VGCCs both in the absence and presence of L-NAME, providing insight into the modulatory role of NO. The AUC was expressed in arbitrary units.

### 2.3 Gene expression analysis

For gene expression analysis, renal vascular tissue was obtained from branches of the main renal artery entering the kidney to ensure a sufficient tissue pool for the experimental protocol.

#### 2.3.1 Total RNA extraction and quality control

Intrarenal arteries were carefully isolated using RNase-free tools (treated with RNaseZap®, Ambion), immediately immersed in 300 μL of Trizol solution (Thermo Fisher Scientific) to stabilize the mRNA, and then snap-frozen and stored at −80 °C. Total RNA was subsequently extracted following the Chomczynski and Sacchi method (Chomczynski and Sacchi, 1987). After mechanical homogenization, the aqueous phase was separated by adding 200 μL of chloroform per 1 mL of Trizol. RNA was precipitated overnight at −20 °C using isopropanol and glycogen. The resulting pellet was washed with ethanol and resuspended in PCR-grade water. RNA concentration and purity were assessed using a NanoDrop spectrophotometer. Samples were considered acceptable if the 260/280 ratio was between 1.8–2.1 and the 260/230 ratio was between 2.0–2.2.

#### 2.3.2 Reverse transcription and real-time PCR (RT-PCR)

Complementary DNA (cDNA) was synthesized from total RNA using the High-Capacity cDNA Reverse Transcription Kit (Thermo Fisher Scientific). Gene expression quantification was performed by RT-PCR using TaqMan® Gene Expression Assays (Applied Biosystems, Foster City, CA) on a QuantStudio 5 Real-Time PCR System (Thermo Fisher Scientific). Target genes included *Ca*_*V*_ *3.1* (Rn01299126_m1), *Ca*_*V*_ *3.2* (Rn01460348_m1), and *Nos3* (Rn07312037_g1), normalized to the housekeeping gene *Gapdh* (Rn01775763_g1). Relative gene expression was calculated using the 2^−ΔΔCt^ method (Livak and Schmittgen, 2001), with all samples run in triplicate and the male WKY group serving as the control.

### 2.4 Measurement of reactive oxygen species (ROS) production by flow cytometry

Intracellular ROS levels in leukocyte populations were quantified using a FACSAria III cytometer (BD Biosciences, ref. 567799) and analysed with BD FACS DIVA 8.0 and FLOWJO V.10.1 software. Whole blood samples were labeled with Brilliant Violet 421™ anti-rat CD45 conjugate (BD Biosciences), and erythrocytes were lysed using eBioscience™ 10x erythrocyte lysis buffer (Invitrogen, Thermo Fisher Scientific, ref. 00-4300-54).

Specific fluorochromes were utilized for ROS detection: DAF-FM Diacetate (1 μM; λ_ex_ 488 nm λ_em_ 530/20 nm excitation and emission wavelengths respectively; Thermo Fisher Scientific, ref. D23841) for nitric oxide (NO) (Díez et al., 2010; Lampiao et al., 2006), Dihydrorhodamine 123 (DHR 123; 100 μM; λ_ex_ 488 nm λ_em_ 530/20 nm, excitation and emission wavelengths, respectively; Thermo Fisher Scientific, ref. D632) for general ROS (H_2_O_2_, ONOO^−^) (Crow, 1997; Emmendörffer et al., 1990), and dihydroethidium (DHE; 2.5 μg/mL; λ_ex_ 561 nm λ_em_ 586/15 nm, excitation and emission wavelengths, respectively; Thermo Fisher Scientific, ref. D1168) for superoxide (O_2_^−^) (Kalyanaraman, 2011). Samples were incubated with the respective fluorochrome at 37 °C for 30 min in the dark. Fluorescence was acquired using appropriate laser and filter settings. Positive controls included NOR-1 (NO generator), tert-butyl hydroperoxide (H_2_O_2_ generator), or plumbagin (O_2_^−^ inducer). A minimum of 10,000 cells were acquired per sample (Supplementary Figure S2).

### 2.5 Statistical analysis

All data are presented as mean ± standard error of the mean (SEM) for blood pressure, leukocyte ROS levels, and vascular reactivity. Vasodilation was expressed as a percentage of relaxation (%) relative to noradrenaline-induced contraction. Vasoconstriction was expressed as a percentage of contraction (%) relative to contraction induced by 60 mM KCl. For concentration-response curves, the negative logarithm of half-maximal effective concentration (pEC_50_) and maximal contraction (E_max_) were calculated. Individual values in bar graphs are represented as scatter dots to show data distribution.

Statistical analyses were performed using GraphPad Prism 9.0.2 (GraphPad Software Inc.). Data normality was assessed using the Shapiro-Wilk, test. Comparisons between groups were assessed using two-way analysis of variance (ANOVA). A *p* value <0.05 was considered statistically significant. Statistical significance is denoted in figures as *p < 0.05, **p < 0.01, ***p < 0.001, and ****p < 0.0001.

## 3 Results

### 3.1 Characterization of vascular function in the renal artery of SHR

**FIGURE 1:**
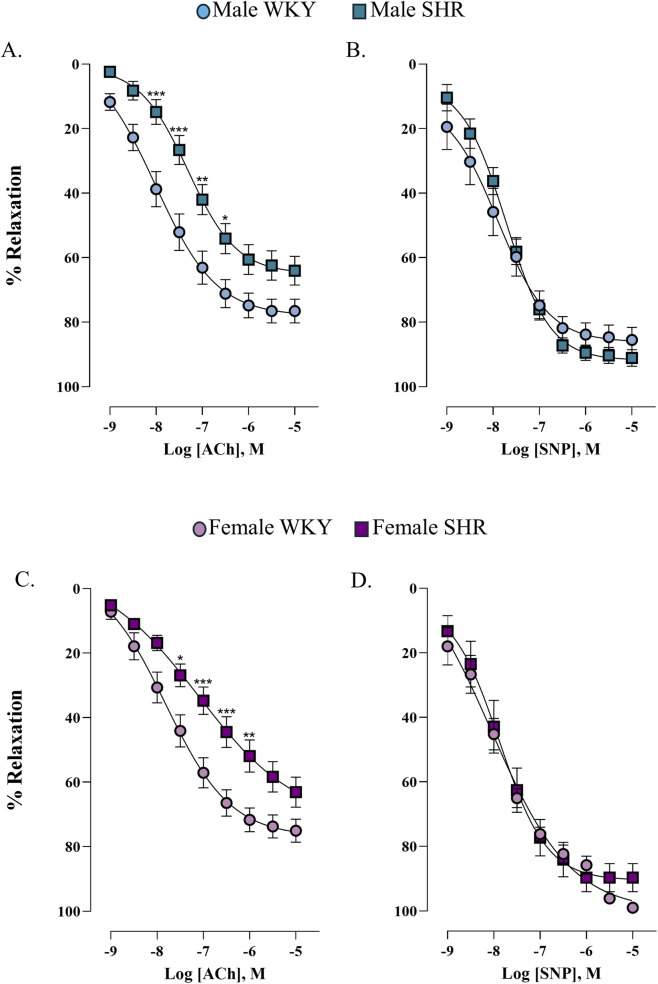
Concentration-response curve to acetylcholine (Ach) and sodium nitroprusside (SNP) in the renal artery of male **(A,B)** and female **(C,D)**. n = 8 (n indicates number of animals). Data are shown as mean ± SEM and were analysed using a two-way ANOVA followed by a Bonferroni *post hoc* test. *p < 0.05; **p < 0.01; ***p < 0.001 compared to WKY group.

To test whether hypertension caused endothelial and vascular dysfunction, concentration-response curves to acetylcholine and sodium nitroprusside were performed in the renal artery of four experimental groups. In our results, SHR exhibited a rightward shift in the acetylcholine curve, indicating endothelial dysfunction in both sexes (Figures 1A,C). Endothelium-independent vasodilation remained intact, as sodium nitroprusside responses were unchanged (Figures 1B,D), indicating that SHR model induces endothelial dysfunction in renal artery. The values for pEC_50_ and E_max_ were summarized in Supplementary Table S1.

**FIGURE 2:**
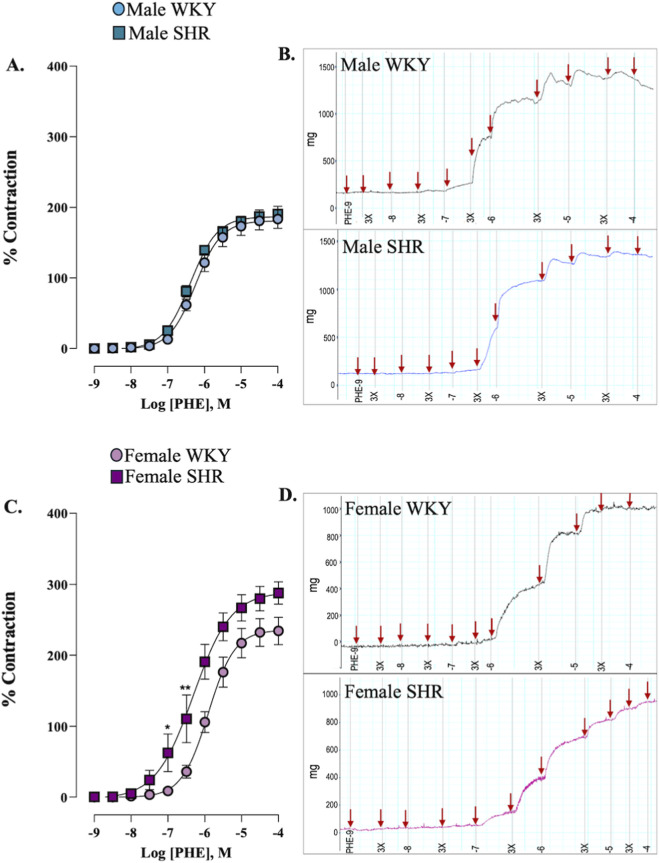
Concentration-response curve to phenylephrine (PHE) in renal artery of male **(A)** and female **(C)** WKY and SHR. Panels **(B)** and **(D)** show representative organ bath traces of PHE-induced contractions in male and female arteries. n = 8 (n indicates number of animals). Data are shown as mean ± SEM and were analysed using a two-way ANOVA followed by a Bonferroni *post hoc* test. *p < 0.05; **p < 0.01 compared to female WKY.

To assess whether hypertension influences α_1_-adrenergic contraction, concentration-response curves to phenylephrine (10^–9^ - 10^–4^ M) were constructed. Our findings indicated that hypertension did not significantly alter the contractile response to phenylephrine in male rats (Figure 2A). Conversely, female SHR exhibited a heightened sensitivity to phenylephrine compared to the WKY group (Figure 2B). This result highlights a significant sex-dependent modulation of vascular sensitivity to α_1_-adrenergic stimulation in the context of hypertension. The values for pEC_50_ and E_max_ were summarized in Supplementary Table S2.

**FIGURE 3:**
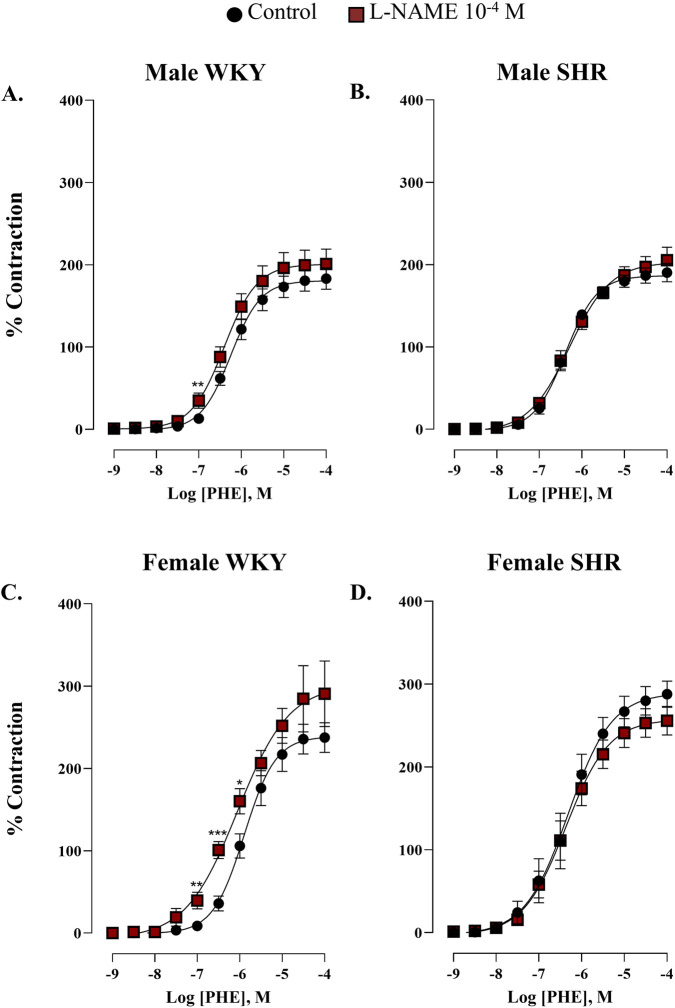
Concentration-response curve to phenylephrine (PHE) in renal artery of male WKY and SHR (**A,B**, respectively) and female WKY and SHR (**C,D**, respectively) groups, in the absence (control) and in the presence of L-NAME (10^−4^ M). n = 7–8 (n indicates number of animals). Data are shown as mean ± SEM and were analysed using a two-way ANOVA followed by a Bonferroni *post hoc* test. *p < 0.05; **p < 0.01; ***p < 0.001 compared to the control curve.

In another set of experiments, concentration-response curves to phenylephrine (10^–9^ - 10^–4^ M) in the presence of L-NAME (10^–4^ M) were performed to evaluate NO release by α_1_-adrenergic stimulation. Our results indicated that hypertension significantly reduced NO production in response to phenylephrine in renal artery in both sexes since incubation with L-NAME did not induce any further change (Figures 3B,D). This impairment was particularly pronounced in hypertensive females, since significant differences were detected only at 10^–7^ M in WKY males (Figure 3A) with and without L-NAME, whereas in WKY females these differences extended to three concentrations (10^–7^, 3 × 10^−7^, and 10^–6^ M) (Figure 3C). The values for pEC_50_ and E_max_ were summarized in Supplementary Table S3.

### 3.2 Involvement of T-type VGCCs in phenylephrine-induced contraction in the renal artery of WKY and SHR groups

**FIGURE 4:**
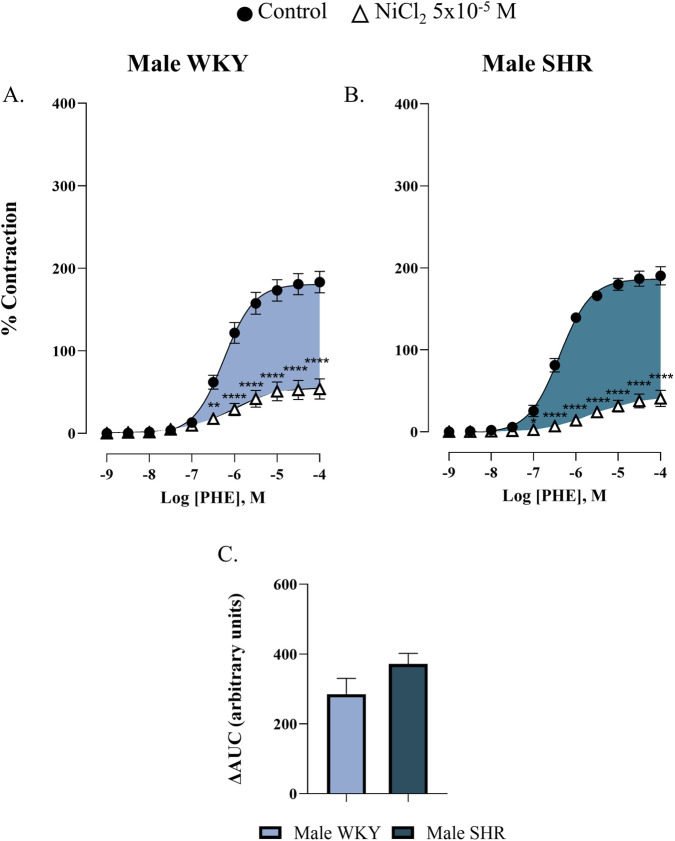
Concentration-response curve to phenylephrine (PHE) in the renal artery of male WKY **(A)** and SHR **(B)** in the absence (control) and presence of nickel chloride (NiCl_2_ 5 × 10^-5^ M). The bar graph **(C)** shows the ΔAUC from PHE curves, indicating the involvement of T-type VGCCs in response to phenylephrine in male WKY (light blue) and SHR (dark blue) groups. n = 8 (n indicates number of animals). Data are shown as mean ± SEM and were analysed using a two-way ANOVA followed by a Bonferroni *post hoc* test. **p < 0.01 ****p < 0.0001 compared to the control curve.

**FIGURE 5:**
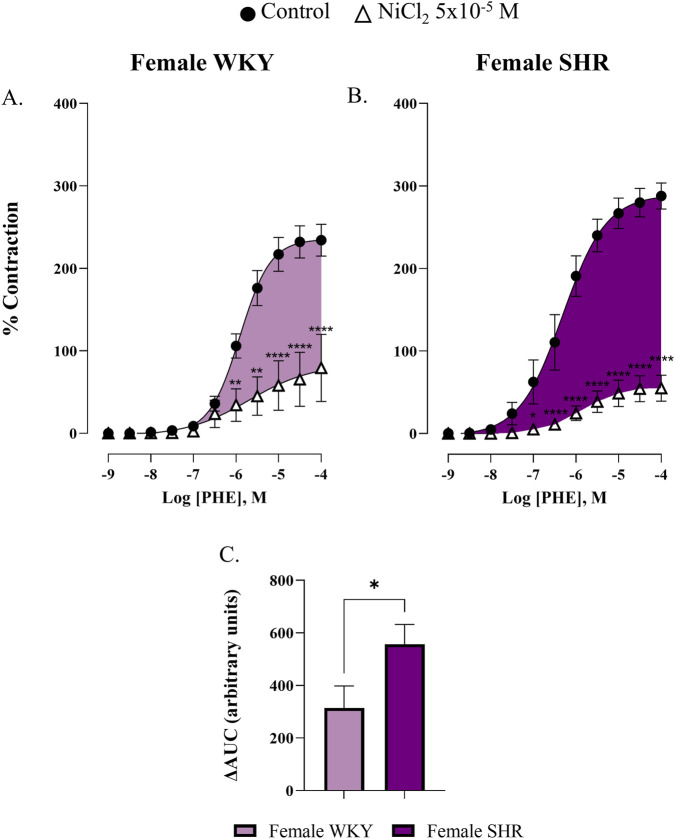
Concentration-response curve to phenylephrine (PHE) in the renal artery of female WKY **(A)** and SHR **(B)** in the absence (control) and presence of nickel chloride (NiCl_2_ 5 × 10^−5^ M). The bar graph **(C)** shows the ΔAUC from PHE curves, indicating the involvement of T-type VGCCs in response to phenylephrine in female WKY (light pink) and SHR (dark pink) groups. n = 7–8 (n indicates number of animals). Data are shown as mean ± SEM and were analysed using a two-way ANOVA followed by a Bonferroni *post hoc* test. *p < 0.05; **p < 0.01 ****p < 0.0001 compared to the control curve.

To study the involvement of T-type VGCCs in the response to phenylephrine, concentration-response curves were performed in the presence of NiCl_2_ (5 × 10^−5^ M). Our results demonstrate that T-type VGCCs contributed to phenylephrine-induced contraction in the renal artery of the four studied groups (Figures 4, 5; Supplementary Table S4). To determine this involvement, the ΔAUC was calculated by subtracting the AUC of the phenylephrine curve incubated with NiCl_2_ from the control phenylephrine curve, as detailed in the Materials and Methods section. The ΔAUC values were 285.1 ± 44.9 a.u. and 371.7 ± 30.6 a.u. for male WKY and male SHR respectively, *p* = 0.1142 (Figure 4C), indicating that hypertension did not increase the participation of T-type VGCCs in this response in males. However, in females, the participation of T-type VGCCs was significantly greater in the SHR group compared to the WKY group (ΔAUC = 314.1 ± 84.2 a.u. for female WKY versus ΔAUC = 556.7 ± 75.5 a.u. for female SHR, *p* = 0.0336), suggesting that hypertension increased the involvement of these channels in the renal artery of female rats (Figure 5C).

### 3.3 Involvement of T-type VGCCs in phenylephrine-induced contraction in the renal artery of WKY and SHR groups in the absence of NO

To determine if NO modulates T-type VGCCs activity, concentration-response curves to phenylephrine were performed in the presence of L-NAME and NiCl_2_. Results were quantified by calculating the ΔAUC, as described in Materials and Methods section, to measure the participation of T-type VGCCs in the absence (ΔAUC2) and presence of NO (ΔAUC1).

**FIGURE 6:**
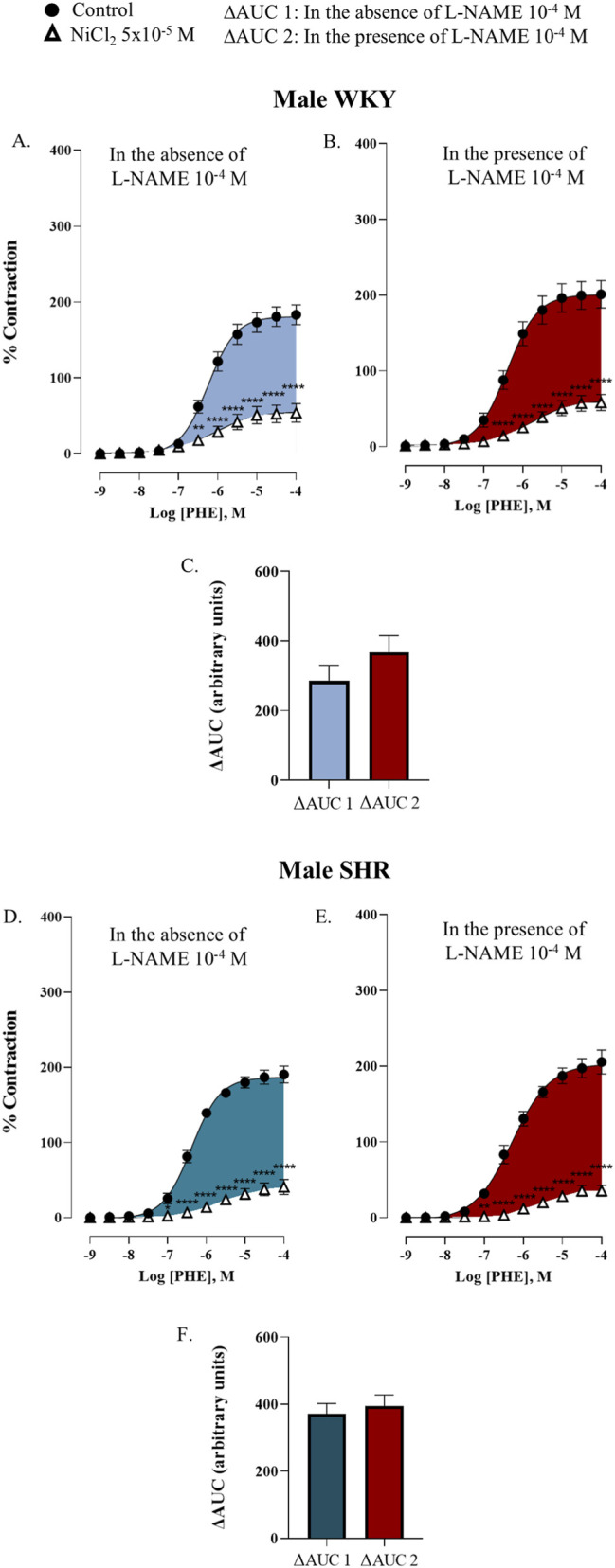
Concentration-response curve to phenylephrine (PHE) in the renal artery of male WKY rat in absence (control) and presence of nickel chloride (NiCl_2_ 5 × 10^−5^ M) **(A)**; in presence of L-NAME (10^–4^ M) (control) and L-NAME (10^–4^ M) plus nickel chloride (NiCl_2_ 5 × 10^−5^ M) **(B)**. Participation of T-type VGCCs in the response to PHE in the absence (ΔAUC1) and presence of L-NAME (10^–4^ M) (ΔAUC2) represented as bars **(C)**; and male SHR in absence (control) and presence of nickel chloride (NiCl_2_ 5 × 10^−5^ M) **(D)**; in presence of L-NAME (10^–4^ M) (control) and L-NAME (10^–4^ M) plus nickel chloride (NiCl_2_ 5 × 10^−^5 M) **(E)**. Participation of T-type VGCCs in the response to PHE in the absence (ΔAUC1) and presence of L-NAME (10^–4^ M) (ΔAUC2) represented as bars **(F)**. n = 8 (n indicates number of animals). Data are shown as mean ± SEM and were analysed using a two-way ANOVA followed by a Bonferroni *post hoc* test. **p < 0.01; ****p < 0.0001 compared to control curve.

In male WKY rats, NOS inhibition with L-NAME did not significantly alter the blocking effect of NiCl_2_ on phenylephrine-induced contraction. The ΔAUC2 was 366.9 ± 48.7 a.u., which did not differ significantly from the ΔAUC1, 285.1 ± 45.0 a.u., *p =* 0.2168 (Figures 6A–C). Similarly, in male SHR, no differences were found between ΔAUC2 and ΔAUC1 (394.5 ± 33.1 a.u. versus 371.7 ± 30.7 a.u., *p =* 0.6143) (Figures 6D–F).

**FIGURE 7:**
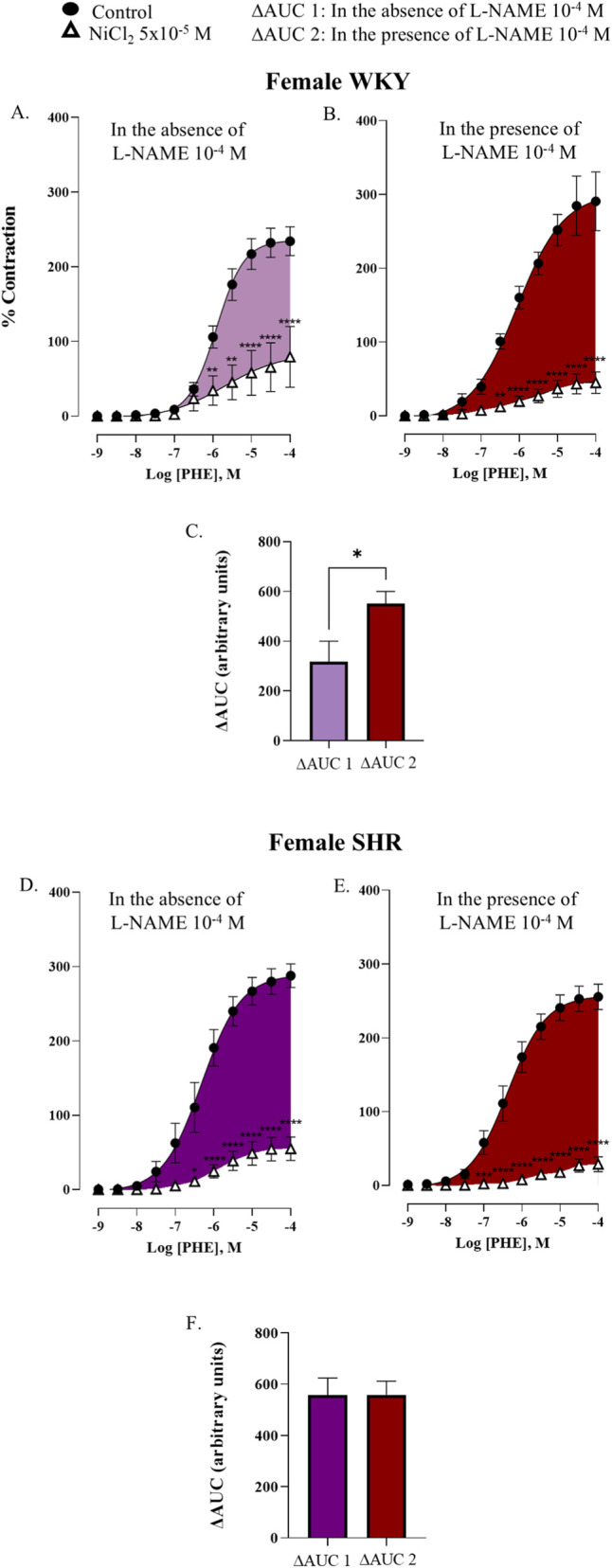
Concentration-response curve to phenylephrine (PHE) in the renal artery of female WKY rat in absence (control) and presence of nickel chloride (NiCl_2_ 5 × 10^−5^ M) **(A)**; in presence of L-NAME (10^–4^ M) (control) and L-NAME (10^–4^ M) plus nickel chloride (NiCl_2_ 5 × 10^−5^ M) **(B)**. Participation of T-type VGCCs in the response to PHE in the absence (ΔAUC1) and presence of L-NAME (10^–4^ M) (ΔAUC2) represented as bars **(C)**; and male SHR in absence (control) and presence of nickel chloride (NiCl_2_5 × 10^−5^ M) **(D)**; in presence of L-NAME (10^–4^ M) (control) and L-NAME (10^–4^ M) plus nickel chloride (NiCl_2_5 × 10^−5^ M) **(E)**. Participation of T-type VGCCs in the response to PHE in the absence (ΔAUC1) and presence of L-NAME (10^–4^ M) (ΔAUC2) represented as bars **(F)**. n = 8 (n indicates number of animals). Data are shown as mean ± SEM and were analysed using a two-way ANOVA followed by a Bonferroni *post hoc* test. **p < 0.01; ****p < 0.0001 compared to control curve.

In female WKY rats, NO inhibition with L-NAME significantly potentiated the blocking action of NiCl_2_. ΔAUC2 was significantly higher than ΔAUC1 (551.1 ± 49.4 a. u. versus 316.8 ± 83.4 a.u. for ΔAUC2 and ΔAUC1, respectively, *p =* 0.0174) (Figures 7A–C). This effect was notably absent in the female SHR group (values were 516.1 ± 62.9 a.u. and 556.7 ± 75.5 a.u. for ΔAUC2 and ΔAUC1, respectively, *p =* 0.9821) (Figures 7D–F), which is consistent with their reduced NO release in response to phenylephrine (Figure 3D). The values for pEC_50_ and E_max_ were summarized in Supplementary Table S5.

Taken together, these findings indicate that hypertensive females exhibit the highest T-type channel activity of the four experimental groups, likely due to the more pronounced reduction in NO release induced by hypertension in this group.

### 3.4 Gene expression analysis of T-type VGCCs and eNOS in intrarenal arteries

**FIGURE 8:**
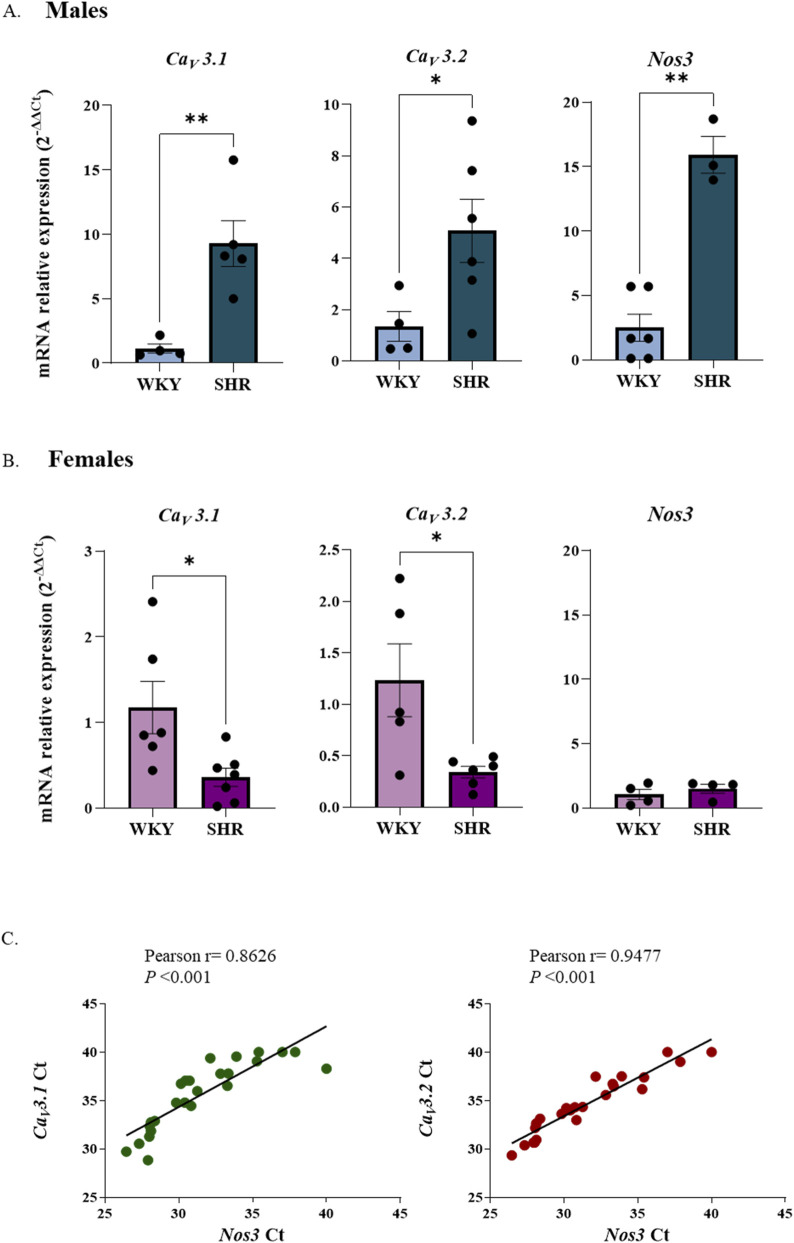
Relative mRNA expression of Ca_V_3.1, Ca_V_3.2 and Nos3 genes in intrarenal arteries of male **(A)** and female **(B)** WKY and SHR groups. n = 6–8 (n indicates number of animals). Correlation between Ct of T-type VGCCs subtypes (Ca_V_3.1 and Ca_V_3.2) and Ct of Nos3 in rat intrarenal arteries (male and female, WKY and SHR groups) **(C)**. Data are shown as mean ± SEM and individual values are represented as scatter dots to show data distribution. The full dataset for each gene was analysed using a two-way ANOVA and Bonferroni *post hoc* test. *p < 0.05; **p < 0.01 compared to WKY group.

To further elucidate the molecular mechanisms, we quantified the gene expression of the predominant vascular T-type VGCC subtypes, (*Ca*_*V*_ *3.1* and *Ca*_*V*_ *3.2*) and *Nos3* (encoding eNOS) in isolated intrarenal arteries from male and female WKY and SHR rats using RT-PCR. The results are summarized in Figure 8.

In male rats, *Ca*_*V*_ *3.1* and *Ca*_*V*_ *3.2* mRNA expression levels were significantly higher in SHR compared to WKY rats (*p =* 0.0090 for *Ca*_*V*_ *3.1* and *p =* 0.0293 for *Ca*_*V*_ *3.2*). Additionally, *Nos3* expression was also elevated in SHR compared with WKY rats (*p =* 0.0013) (Figure 8A).

In contrast, among females, *Ca*_*V*_ *3.1* and *Ca*_*V*_ *3.2* mRNA levels were significantly lower in SHR compared to WKY rats (*p =* 0.0439 for *Ca*_*V*_ *3.1* and *p =* 0.0229 for *Ca*_*V*_ *3.2*). *Nos3* expression remained unchanged between female SHR and WKY groups (Figure 8B).

Furthermore, a positive correlation was observed between the Ct values of *Ca*_*V*_ *3.1* and *Nos3*, as well as between *Ca*_*V*_ *3.2* and *Nos3* (Figure 8C).

### 3.5 Flow cytometric analysis of oxidative stress markers in circulating leukocytes and neutrophils

**FIGURE 9:**
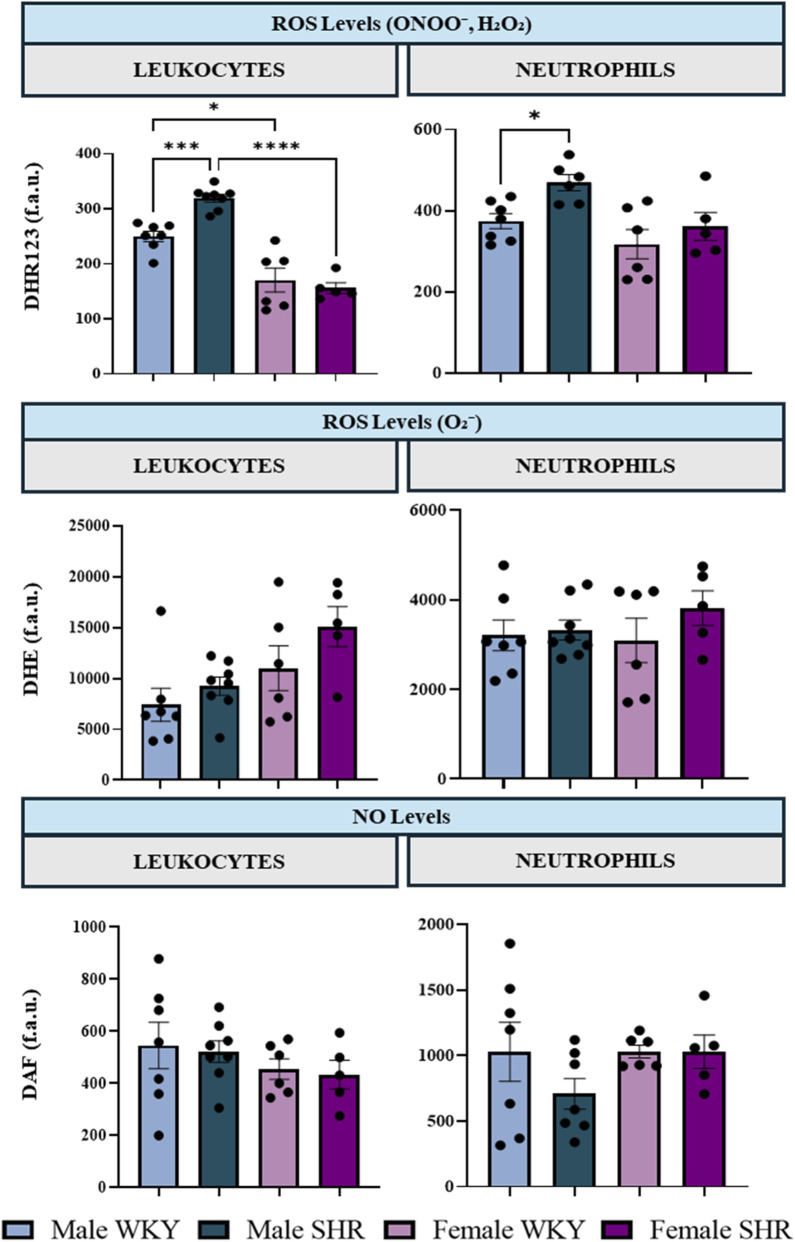
Bar graphs summarising levels of H_2_O_2_, ONOO^−^ and O_2_^−^ as well as intracellular NO in leukocytes and neutrophils, measured by using the fluorescence markers DHR 123, DHE and DAF, respectively. n = 6–8 per group (n indicates number of animals). Bars represent mean ± SEM and individual values are represented as scatter dots to show data distribution. Data were analysed using a two-way ANOVA followed by a Bonferroni *post hoc* test. *p < 0.05, **p < 0.01, ***p < 0.001, and ****p < 0.0001.

To assess whether the vascular tissue of SHR was exposed to higher levels of oxidative stress, we quantified basal intracellular ROS levels in circulating white blood cells. Our findings demonstrated that male SHR exhibited significantly increased production of H_2_O_2_ and ONOO^−^ in both total leukocytes and neutrophils compared to WKY rats as evidenced by the greater DHR123 fluorescence signal (Figure 9). Moreover, male SHR exhibited higher DHR123 fluorescence compared with female SHR, suggesting that the vascular environment in males may be subject to greater oxidative stress than in WKY or female SHR. Although these measurements reflect systemic oxidative status, specifically in circulating leukocytes, they indicate that hypertension induces a more pronounced oxidative burden in male leukocytes relative to females, providing relevant insights into the model used in our study.

## 4 Discussion

The present study follows directly from our recent work (Suarez et al., 2026), in which we demonstrated a functional role for T-type VGCCs in rabbit arteries. That study established the involvement of T-type channels in the regulation of vascular tone and demonstrated their dual role in the renal artery: facilitating endothelial NO generation, while NO, in turn, acts as a negative regulator of T-type VGCC-mediated phenylephrine-induced contraction. In the current study, we aim to expand this knowledge and investigate the role of these channels in hypertension and their sex-specific differences.

The renal system is pivotal in long-term blood pressure regulation, controlling fluid volume, sodium excretion, and neurohumoral mechanisms (Guyton et al., 1972; Ivy and Bailey, 2014; Wenner et al., 2018). While primarily a conduit, the renal artery is a significant site for vascular pathology in hypertension, as its dysfunction can directly impact both renal perfusion and systemic blood pressure (Sos and Trost, 1995). Within the renal microvasculature, L-type VGCCs are predominantly located in afferent arterioles, while T-type channels are functionally expressed in both afferent and efferent arterioles (Feng et al., 2004; Homma et al., 2013). Studies such as those by Hansen et al. demonstrate the expression and functional activity of these channels in renal cortical preglomerular vessels, juxtamedullary efferent arterioles, and the outer medullary vasa recta, but not in cortical efferent arterioles (Hansen et al., 2001). However, despite this distinct distribution, T-type VGCCs may not be essential for the renal autoregulation in response to acute increases in renal perfusion pressure (Frandsen et al., 2016). Overall, the distinct expression and functional profile of T-type VGCCs points to a specialized role in regulating renal hemodynamic.

Evidence from other vessels indicates that T-type VGCCs play a significant role in controlling vascular resistance and myogenic tone (VanBavel et al., 2002; El-Rahman et al., 2013; Navarro-Gonzalez et al., 2009; Braunstein et al., 2009; Gustafsson et al., 2001). In intraseptal coronary artery, T-type VGCCs act as the electrophysiological mechanism that translates NO loss into vascular smooth muscle hyperexcitability, leading to vasoconstriction and vasospasm, particularly when the protective DDAH1 system is compromised (Ng et al., 2024). In mesenteric arteries, T-type VGCCs also contribute to the generation of transient depolarizing spikes and vasospasm following the loss of endothelial NO (Smi et al., 2020). These studies reinforce our previous observations that T-type VGCCs exhibit higher activity in the renal artery under conditions of NO inhibition, compared with other conduit arteries (Suarez et al., 2026), underscoring their function as critical modulators of renal vascular tone.

It is increasingly recognized that sex is a crucial biological variable influencing cardiovascular physiology and pathology, including responses to hypertension and calcium channel activity (Niyonsaba et al., 2015). Hypertension is well-established in the SHR model (Doris, 2017; Butts et al., 2022; Hohl et al., 2023; Safar et al., 2001). At 16 weeks of age, SHR exhibit stable hypertension together with well-documented endothelial dysfunction (Leal et al., 2020). Importantly, vascular alterations in this model are mainly related to reduced nitric oxide bioavailability (Zalba et al., 2001). These characteristics make the SHR particularly suitable for investigating NO-dependent vascular mechanisms. Consistent with the literature, our results confirmed significant increases in systolic, diastolic, and mean arterial pressure in both male and female SHR compared to WKY rats of the same sex, as well as an endothelial dysfunction, evidenced by a rightward shift of the acetylcholine-mediated vasorelaxation curve.

To further characterize the hypertensive milieu, we assessed basal intracellular levels of ROS in circulating leukocytes. Our data revealed that H_2_O_2_ and ONOO^−^ production was significantly increased in male SHR, aligning with numerous studies describing elevated oxidative stress in male SHR across various tissues (Cerecedo et al., 2023; Cuzzocrea et al., 2004; Randriamboavonjy et al., 2017; Souza Bomfim et al., 2022; Wu et al., 2023). In contrast, we observed no significant differences in oxidative stress in females between the SHR and WKY groups, strongly suggesting a protective effect of the female sex against hypertension-induced systemic oxidative stress (Gillis et al., 2018; Sartori-Valinotti et al., 2007; Sullivan et al., 2007). A limitation of this study is that oxidative stress was evaluated in blood cells as a surrogate marker of systemic redox status, rather than being directly assessed in vascular tissue. Consequently, the lack of detectable changes in blood cell ROS in female SHR does not rule out enhanced oxidative stress at the vascular wall, where localized ROS production may impair NO bioavailability without markedly altering systemic oxidative indices.

Sex-specific differences in NO bioavailability have been reported, indicating that females often exhibit enhanced NO production compared to males, potentially due to the modulatory effects of oestrogens (Elmarakby et al., 2023). Our results show that hypertension increased sensitivity to phenylephrine only in female rats, where NO plays a more prominent modulatory role in α_1_-adrenergic-mediated vasoconstriction compared to males. It is well established that NO deficiency is a key contributor to the progression and maintenance of hypertension in SHR (Anishchenko et al., 2015) and that its restoration mitigates vascular impairment (Lataro et al., 2019); however, most studies have not addressed sex differences. Our results show that the loss of NO is more pronounced in female than in male hypertensive rats.

T-type VGCCs participate in phenylephrine-induced contraction in the renal arteries of both male and female rats, and hypertension selectively increased the involvement of T-type VGCCs in female rats only, highlighting a previously unrecognized sex-dependent modulation of these channels in vascular dysfunction. Building upon previous studies suggesting that T-type VGCCs are upregulated under conditions of reduced NO bioavailability (Ng et al., 2024; Smi et al., 2020; Howitt et al., 2015; Howitt et al., 2013b), we investigated their functional role under pharmacological inhibition of NO synthesis. In male WKY and SHR groups, L-NAME did not augment the NiCl_2_ blockade, suggesting T-type VGCCs function is neither significantly upregulated in the absence of NO, nor substantially affected by hypertension in males. However, it should be noted that the WKY male group already exhibited limited NO release in response to phenylephrine, with only a single concentration showing significant differences across the curve, resulting in a minimal modulatory effect of NO on T-type channels in males. In females, L-NAME significantly increased the functional contribution of T-type VGCCs in WKY rats, indicating that physiological NO exerts an inhibitory modulation over these channels. This enhanced T-type VGCCs activity in the presence of L-NAME was abolished in female SHR, consistent with their already reduced NO release in response to phenylephrine. These findings suggest that in hypertensive females, T-type VGCCs become more active due to compromised NO bioavailability. These findings align with emerging evidence indicating that calcium channel blockers may be more effective in hypertensive females, as supported by recent computational modelling and a systematic review and meta-analysis (van Luik et al., 2023; Hernandez-Hernandez et al., 2024).

At the gene expression level, male SHR exhibited significantly upregulated mRNA levels of *Ca*_*V*_ *3.1* and *Ca*_*V*_ *3.2* and *Nos3* compared to WKY rats. This finding may be related to oxidative stress, as T-type VGCCs become more active under conditions of increased ROS (Howitt et al., 2013a). Therefore, the more oxidative environment observed in male SHR, as evidenced by the higher DHR123 fluorescence detected by flow cytometry, may contribute to the increased mRNA expression of T-type VGCCs. However, the precise functional relationship between oxidative stress and Ca_V_3 activity remains complex and potentially contradictory. While some studies suggest increased activity under certain ROS conditions (Howitt et al., 2013a), a recent *patch clamp* study on recombinant *Ca*_*V*_ *3*.2 channels demonstrated that extracellular redox agents and ROS production inhibit the current (Huang et al., 2020). Moreover, *Nos3* gene expression was also significantly higher in male SHR compared to WKY rats, which may represent a compensatory response to elevated oxidative stress (Kunsch and Medford, 1999; Zhen et al., 2008). Despite the hypertension-induced overexpression of *Ca*_*V*_ *3.1*, *Ca*_*V*_ *3.2,* and *Nos3* in the intrarenal arteries of male rats, no corresponding increase in the functional involvement of T-type VGCCs or enhanced NO release was observed in the renal artery. These findings suggest that the upregulated mRNA expression we observed may represent a compensatory mechanism, an attempt by the vascular cells to restore the function in an environment of oxidative stress, rather than a direct cause of activation. To date, no published evidence directly supports that oxidative stress increases the steady-state gene or protein expression of these channels in the vasculature. Therefore, we hypothesize that our findings may be attributable to a transcriptional upregulation associated with increased oxidative stress; however, additional studies will be necessary to elucidate this possibility.

Conversely, in female SHR, *Ca*_*V*_ *3.1* and *Ca*_*V*_ *3.2* mRNA levels were significantly lower compared to female WKY. This reduced expression paradoxically does not translate into reduced functional activity in the renal artery; instead, we found an increased involvement of these channels in female SHR. This intriguing discrepancy might suggest that increased T-type VGCCs activity in hypertensive females exerts inhibitory feedback on *Ca*_*V*_ *3.1* and *Ca*_*V*_ *3.2* gene expression. Hypertension did not significantly alter *Nos3* gene expression in females, indicating an inability of hypertensive females to upregulate *Nos3*. Other authors have also observed that eNOS gene expression is not modified in hypertensive females (Franco et al., 2002), despite the presence of endothelial dysfunction.

The positive correlation between *Ca*_*V*_ *3.1*, *Ca*_*V*_ *3.2*, and *Nos3* gene expression could support the idea of functional crosstalk between these channels and eNOS. Indeed, T-type VGCCs transcripts and proteins (Ca_V_3.1 and Ca_V_3.2) have been identified in the smooth muscle cells of various vascular beds and species (El-Rahman et al., 2013; Braunstein et al., 2009; Björling et al., 2013; Harraz et al., 2015) and in the endothelium (Braunstein et al., 2009; Kuo et al., 2010; Mikkelsen et al., 2016), where Ca_V_3.1 has been shown to colocalize with eNOS (Suarez et al., 2026; Svenningsen et al., 2014). The differential distribution of T-type VGCCs among vessels suggests that their contribution to vascular tone regulation is closely conditioned by the type of vessel and the cellular compartment in which they are expressed. For instance, in the rabbit aorta, Ca_V_3.1 is predominantly located in the endothelium, with no detectable signal in the smooth muscle layer, while Ca_V_3.2 is expressed in both endothelial cells and vascular smooth muscle, which is consistent with a predominantly contractile function (Suarez et al., 2026). In contrast, in the rabbit renal artery, the concomitant expression of Ca_V_3.1 and Ca_V_3.2 with a punctate distribution in both the endothelium and the tunica media, together with the clear colocalization of Ca_V_3.1 with eNOS, supports the existence of Ca^2+^-NO signalling microdomains and this probably facilitate endothelial NO-dependent vasodilation (Suarez et al., 2026). This functional coupling reinforces the idea that T-type VGCCs, far from playing a uniform role, participate in a differentiated manner in the integration of contractile and relaxant signals depending on the vascular territory, being especially relevant in renal circulation (Suarez et al., 2026). Further research is warranted to elucidate this crosstalk.

An important methodological consideration is the use of nickel chloride as a pharmacological inhibitor of T-type VGCCs. Although nickel can produce off-target effects after long-term inhalation exposure such as endothelial dysfunction or effects on vascular function beyond ion channel inhibition (Ying et al., 2013; Wani et al., 2018), its selectivity is highly concentration dependent. Extensive evidence indicates that, at low micromolar concentrations, nickel chloride preferentially inhibits T-type VGCCs, with Caᵥ3.2 being the most sensitive subtype. Reported IC_50_ values for Caᵥ3.2 lie in the low micromolar range (∼10–15 µM), whereas Caᵥ3.1 is markedly less sensitive (IC_50_ ∼200–300 µM) (Harraz et al., 2013; Harraz et al., 2014). In contrast, high-voltage–activated L-type Caᵥ1.2 channels require millimolar concentrations (Lacinová, 2011; Lee et al., 1999). Consistent with this profile, Smith et al. (2020) reported that 50 µM nickel did not affect transient spikes or vasoconstriction evoked by the Caᵥ1.2 activator Bay K-8644 in small arteries, supporting the lack of functional inhibition of L-type channels at this concentration and suggesting a predominant contribution of Caᵥ3.2 VGCCs. These considerations indicate that the effects observed in our study largely reflect selective inhibition of Caᵥ3.2 rather than nonspecific blockade of VGCCs or off-target vascular effects.

In summary, our findings reveal sex-dependent modulation of T-type VGCCs in the renal artery during hypertension. While both sexes develop endothelial dysfunction, hypertensive females exhibit enhanced T-type VGCC activity and more severe NO loss in response to phenylephrine, whereas males display oxidative stress and transcriptional upregulation of T-type channels without functional consequences. These results underscore the importance of incorporating sex as a biological variable in hypertension research and highlight T-type VGCCs as potential therapeutic targets, particularly in women.

## Data Availability

The raw data supporting the conclusions of this article will be made available by the authors, without undue reservation.

## Supplementary material

SUPPLEMENTARY FIGURE S1 Systolic, diastolic, mean and pulse pressure (mmHg) values of male and female WKY and SHR groups. n = 8 per group (n indicates number of animals). Bars represent mean ± SEM and individual values are represented as scatter dots to show data distribution. Data were analysed using a two-way ANOVA followed by a Bonferroni post hoc test. ***p < 0.001 and ****p < 0.0001.

SUPPLEMENTARY FIGURE S2 Flow cytometry gating strategy. (A) FSA vs FSH dot plot shows the selection of singlet cells. In the CD45 vs SS A dot plot, singlet cell gating is applied. The leukocyte, lymphocyte+monocyte, and neutrophil regions are determined based on the fluorescence intensity for CD45. In the following three dot plot, these populations are selected, and the fluorescence intensity (Fluorescence Arbitrary Units, FAU) of the fluorochrome DHR123 was quantified in relation to the intracellular level of hydrogen peroxide and hydroxyl ions. (B) FSA vs FSH dot plot shows the selection of singlet cells. In the CD45 vs SS A dot plot, singlet cell gating is applied. The leukocyte, lymphocyte+monocyte, and neutrophil regions are determined based on the fluorescence intensity for CD45. In the following three dot plot, these populations are selected, and the fluorescence intensity (Fluorescence Arbitrary Units, FAU) of the fluorochrome DAF was quantified in relation to the intracellular level of nitric oxide. (C) FSA vs FSH shows the selection of singlet cells. In the CD45 vs SS A dot plot, singlet cell gating is applied. The leukocyte, lymphocyte+monocyte, and neutrophil regions are determined based on the fluorescence intensity for CD45. In the following three histograms, these populations are selected, and the fluorescence intensity (Fluorescence Arbitrary Units, FAU) of the fluorochrome DHE is quantified in relation to the intracellular level of superoxide.
