# Supplementary material for: Sex-specific modulation of T-type voltage-gated calcium channels in the renal artery of hypertensive rats
Source: Front Physiol. 2026 Mar 16;17:1754344. doi: 10.3389/fphys.2026.1754344 (PMC13033523; doi:10.3389/fphys.2026.1754344)
Supplement: Supplementary file 1 [file Table1.docx]

*Supplementary Table S1*. *Values of pEC50 ± standard error of the mean (SEM) and Emax ± SEM (expressed as a percentage of contraction (%) relative to contraction induced by 60 mM KCl) of concentration-response curves to acetylcholine and sodium nitroprusside in the renal artery of male and female WKY and SHR groups.*

| **Acetylcholine** | **n** | **pEC50 ± SEM** | **Emax ± SEM (%)** |  |
| --- | --- | --- | --- | --- |
| Male WKY | 8 | 7.81 ± 0.12 | 78.83 ± 3.36 |  |
| Male SHR | 8 | 7.23 ± 0.12* | 64.26 ± 7.23* |  |
| Female WKY | 7 | 7.62 ± 0.12 | 73.22 ± 3.53 |  |
| Female SHR | 7 | 7.16 ± 0.09* | 70.03 ± 3.75* |  |
| **Sodium Nitroprusside** | **n** | **pEC50 ± SEM** | **Emax ± SEM (%)** |  |
| Male WKY | 8 | 8.26 ± 0.24 | 85.45 ± 3.81 |  |
| Male SHR | 8 | 7.89 ± 0.13 | 91.08 ± 2.53 |  |
| Female WKY | 7 | 7.96 ± 0.05 | 86.66 ± 3.27 |  |
| Female SHR | 7 | 7.93 ± 0.16 | 91.38 ± 4.12 |  |

*n= number of animals. *p<0.05 compared to male WKY.*
