# Supplementary figures and images for: Sex-specific modulation of T-type voltage-gated calcium channels in the renal artery of hypertensive rats
Source: Front Physiol. 2026 Mar 16;17:1754344. doi: 10.3389/fphys.2026.1754344 (PMC13033523; doi:10.3389/fphys.2026.1754344)

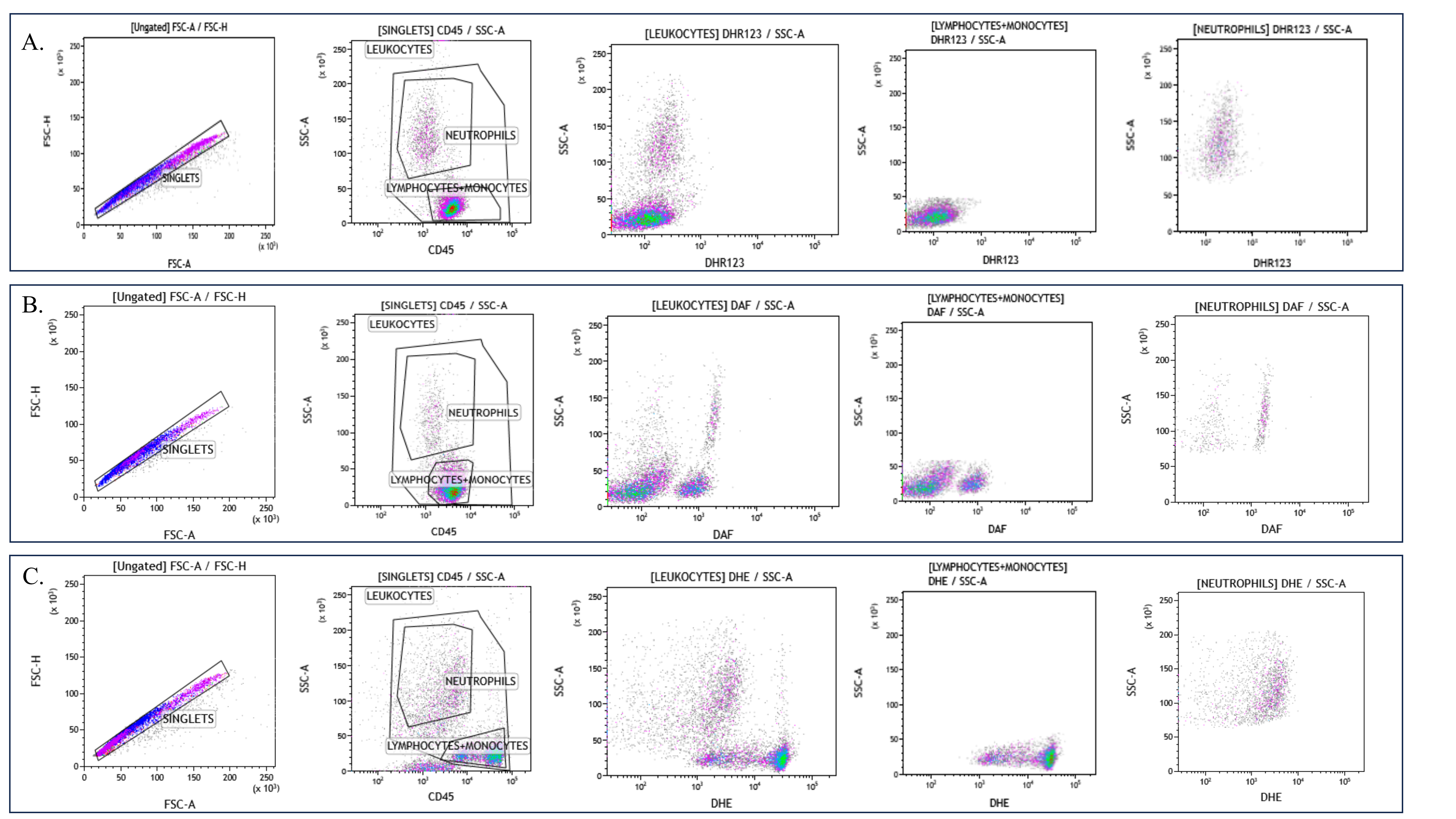

Supplement: Supplementary file 2 [file Image2.tif]

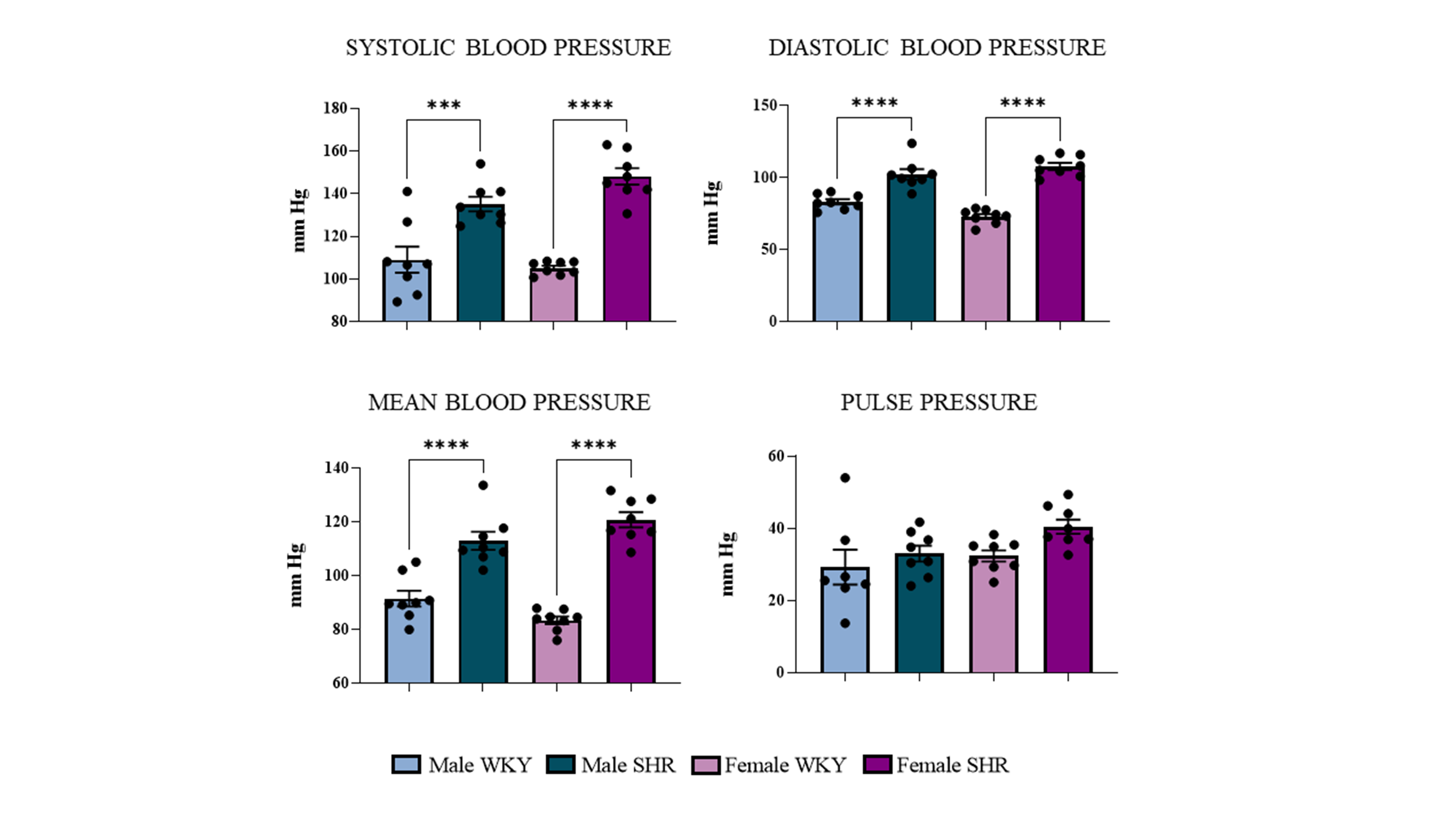

Supplement: Supplementary file 3 [file Image1.tif]
