# Supplementary material for: Sex-specific modulation of T-type voltage-gated calcium channels in the renal artery of hypertensive rats
Source: Front Physiol. 2026 Mar 16;17:1754344. doi: 10.3389/fphys.2026.1754344 (PMC13033523; doi:10.3389/fphys.2026.1754344)
Supplement: Supplementary file 4 [file Table2.docx]

*Supplementary Table S2*. *Values of pEC50 ± standard error of the mean (SEM) and Emax ± SEM (expressed as a percentage of contraction (%) relative to contraction induced by 60 mM KCl) of concentration-response curves to phenylephrine in the renal artery of male and female WKY and SHR groups.*

| **Phenylephrine** | **n** | **pEC50 ± SEM** | **Emax ± SEM**  **(%)** |
| --- | --- | --- | --- |
| Male WKY | 8 | 6.26 ± 0.10 | 183.17 ± 13.17 |
| Male SHR | 8 | 6.43 ± 0.07 | 190.33 ± 11.03 |
| Female WKY | 8 | 5.84 ± 0.08 | 237.50 ± 17.99 |
| Female SHR | 8 | 6.42 ± 0.12* | 287.75 ± 15.84 |

*n= number of animals. *p<0.05 compared to female WKY.*
