# Supplementary material for: Sex-specific modulation of T-type voltage-gated calcium channels in the renal artery of hypertensive rats
Source: Front Physiol. 2026 Mar 16;17:1754344. doi: 10.3389/fphys.2026.1754344 (PMC13033523; doi:10.3389/fphys.2026.1754344)
Supplement: Supplementary file 5 [file Table5.docx]

*Supplementary Table S5*. *Values of pEC50 ± standard error of the mean (SEM) and Emax ± SEM (expressed as a percentage of contraction (%) relative to contraction induced by 60 mM KCl) of concentration-response curves to phenylephrine in the renal artery of male and female WKY and SHR groups in the presence of L-NAME 10^-4^ M, in the absence and presence of nickel chloride (NiCl2) (5x10^-5^ M).*

| **Phenylephrine** | **n** | **pEC50 ± SEM** | **Emax ± SEM**  **(%)** |
| --- | --- | --- | --- |
| **Male WKY** |  |  |  |
| L-NAME 10^-4^ M | 8 | 6.45 ± 0.07 | 201.13 ± 18.15 |
| L-NAME 10^-4^ M + NiCl2 5x10^-5^ M | 8 | 5.90 ± 0.10* | 62.50 ± 11.95* |
| **Male SHR** |  |  |  |
| L-NAME 10^-4^ M | 8 | 6.32 ± 0.12 | 205.44 ± 15.62 |
| L-NAME 10^-4^ M + NiCl2 5x10^-5^ M | 8 | 5.61 ± 0.11* | 50.40 ± 6.89* |
| **Female WKY** |  |  |  |
| L-NAME 10^-4^ M | 8 | 6.01 ± 0.19 | 290.80 ± 39.81 |
| L-NAME 10^-4^ M + NiCl2 5x10^-5^ M | 8 | 6.00 ± 0.20* | 67.25 ± 21.18* |
| **Female SHR** |  |  |  |
| L-NAME 10^-4^ M | 8 | 6.18 ± 0.08 | 255.78 ± 17.18 |
| L-NAME 10^-4^ M + NiCl2 5x10^-5^ M | 8 | 5.82 ± 0.20* | 40.78 ± 14.87* |

*n= number of animals. *p<0.05 compared to L-NAME 10^-4^ M of the same group.*
