# Supplementary material for: Sex-specific modulation of T-type voltage-gated calcium channels in the renal artery of hypertensive rats
Source: Front Physiol. 2026 Mar 16;17:1754344. doi: 10.3389/fphys.2026.1754344 (PMC13033523; doi:10.3389/fphys.2026.1754344)
Supplement: Supplementary file 7 [file Table4.docx]

*Supplementary Table S4*. *Values of pEC50 ± standard error of the mean (SEM) and Emax ± SEM (expressed as a percentage of contraction (%) relative to contraction induced by 60 mM KCl) of concentration-response curves to phenylephrine in the renal artery of male and female WKY and SHR groups in the absence and presence of nickel chloride (NiCl2) (5x10^-5^ M).*

| **Phenylephrine** | **n** | **pEC50 ± SEM** | **Emax ± SEM**  **(%)** |
| --- | --- | --- | --- |
| **Male WKY** |  |  |  |
| Control | 8 | 6.34 ± 0.08 | 185.20 ± 14.50 |
| NiCl2 5x10^-5^ M | 8 | 6.02 ± 0.12* | 65.90 ± 28.18* |
| **Male SHR** |  |  |  |
| Control | 8 | 6.43 ± 0.07 | 190.33 ± 11.03 |
| NiCl2 5x10^-5^ M | 8 | 5.73 ± 0.12* | 45.02 ± 19.46* |
| **Female WKY** |  |  |  |
| Control | 8 | 5.84 ± 0.08 | 234.14 ± 19.34 |
| NiCl2 5x10^-5^ M | 8 | 5.91 ± 0.24 | 79.25 ± 40.70* |
| **Female SHR** |  |  |  |
| Control | 7 | 6.11 ± 0.15 | 278.29 ± 25.09 |
| NiCl2 5x10^-5^ M | 7 | 5.66 ± 0.17* | 74.25 ± 16.15* |

*n= number of animals. *p<0.05 compared to control of the same group.*
